# Editorial Comment: Comparison of mini percutaneous nephrolithotomy and standard percutaneous nephrolithotomy for renal stones >2cm: a systematic review and meta-analysis

**DOI:** 10.1590/S1677-5538.IBJU.2022.03.02

**Published:** 2022-04-14

**Authors:** Alexandre Danilovic

**Affiliations:** 1 Faculdade de Medicina da Universidade de São Paulo Hospital das Clínicas Departamento de Urologia São Paulo SP Brasil Departamento de Urologia, Hospital das Clínicas, Faculdade de Medicina da Universidade de São Paulo, São Paulo, SP, Brasil

Pengfei Qin 1, Dong Zhang 1, Ting Huang 2, Li Fang 2, Yue Cheng 2

Int Braz J Urol . 2021 Oct 8;47. Online ahead of print.

## COMMENT

Percutaneous nephrolithotomy (PCNL) is the treatment modality for renal stones > 20 mm recommended by the European Association of Urology and by the American Urological Association (1, 2). Sepsis and bleeding are among the most feared complications of PCNL and perhaps one of the causes for this surgery represents less than 5% of all kidney stone treatment modalities in Brazil and in the World (3, 4). Also, standard 30 Fr PCNL may cause infundibular strictures in the entry calyx (5). Attempts to minimize bleeding during PCNL led to technique modifications including reducing the percutaneous tract size to decrease the area of parenchymal and infundibular injury (6, 7).

Percutaneous tract miniaturization evolved to several different techniques according to tract size. Indications for each technique vary according to stone size. Micro-PCNL (4.8-10 Fr) is more appropriate for kidney stones up to 15 mm, ultramini-PCNL (11-13 Fr) suits best for kidney stones up to 20 mm. However, there is no clear consensus about which tract size is best for kidney stones > 20 mm (8).

The systematic review and meta-analysis conducted by Qin et al compared the efficacy and safety of mini (16-20 Fr) versus standard (24-30 Fr) percutaneous nephrolithotomy for renal stones more than 2 cm (9). Authors included seven randomized controlled trials in their meta-analysis, involving 1407 mini-PCNL cases and 1436 standard-PCNL cases. Main finding was that mini-PCNL has a similar stone free rate than standard-PCNL. A subgroup analysis showed no difference in stone free rates between 30 Fr and 24 Fr and mini-PCNL groups. Operation time was shorter in standard-PCNL (both 30 Fr and 24 Fr) than mini-PCNL. Standard-PCNL was associated with more hemoglobin drop and blood transfusion rate than mini-PCNL. However, no significant differences were noted between 24 Fr and mini-PCNL regarding hemoglobin drop and blood transfusion rate. Shorter length of hospitalization was associated with mini-PCNL. No significant difference was noted in fever between groups.

The strength of this study is the inclusion of randomized controlled trials and exclusion of retrospective or case-control studies, whereas limitation is that the role of mini-PCNL in the treatment of staghorn stones or in infected stones was not addressed by the studies included in the meta-analysis. Infected stones are a risk factor for postoperative sepsis. Miniaturization of the percutaneous tract may increase the renal pelvic pressure and absorption of irrigation fluid due to limited outflow (10). Althought this meta-analysis showed no significant difference in postoperative fever between groups, more studies are needed to establish the best percutaneous tract size for staghorn and infectious stones. The meta-analysis presented by Qin et al. supports that standard 24 Fr PCNL is the best option for kidney stones > 20 mm combining same stone free rates than 30 Fr with similar blood loss of mini-PCNL but with shorter operation time (9).

Falahatkar et al. studied the effects of pregabalin, solifenacin and their combination therapy on urinary stent-related symptoms in a randomized controlled clinical trial (17). Patients were randomly allocated into four groups: pregabalin 75 mg BID (N=64), solifenacin 5 mg once a day (N=64), pregabalin 75 mg BID and solifenacin 5 mg once a day (N=64), and no medication (N=64). Ureteral Symptom Score Questionnaire (USSQ) was used to compare groups at 2 and 4 weeks after discharge from hospital (18). Authors reported significant beneficial effects in all indexes of USSQ only for combined pregabalin and solifenacin therapy over control group. Reported side effects were mild for all studied groups. Lack of a placebo arm and application of USSQ only at 2 and 4 weeks after discharge from hospital are some of the limitations of this study.

Urinary stent-related symptoms should not be overlooked and could be relieved by an adequate stent selection and a combination of postoperative medical therapy.
